# Notes and News

**Published:** 1887-05-07

**Authors:** 


### Notes and News.

Collected and Communicated.

We informed our readers some time since that it was intended to invite the Prince of Wales to open the new Nursing Home at the London Hospital in the Whitechapel Road. Both the Prince and the Princess have accepted the invitation, and the ceremony has been fixed to take place on Saturday, 21st inst., at 5 p.m. The new buildings will accommodate 100 nurses.

A generous gift of £50 has been received by the Boston Hospital from Mr. S. Harrison, of 4, Wimpole Street, Cavendish Square, as an earnest of the sympathy he feels for the sick and suffering of his native town of Boston.

The friends of Miss Nutting, who died in the Radcliffe Infirmary, whither she was conveyed after a railway accident, have given £500 for the special endowment of a bed in the female ward of this institution.

Mr. Joseph Ruston, of the well known-firm of Messrs. Ruston and Sons of Lincoln, has been elected vice-president of the County Hospital. A grand bazaar is to be held early in July to assist in clearing off the existing debt, and, if possible, to add to the Jubilee Fund which is now being raised for the permanent endowment of this valuable institution.

A Convalescent Home and Chronic Hospital is about to be erected at Cookridge, as an adjunct to the Leeds Infirmary, thanks to the munificence of Mr. John North, of Leeds. The institution, which is to contain accommodation for forty-two patients, will be a very handsome building 230 feet in length, with a depth of 120 feet. The Home is to be erected from the designs of Messrs. Chorley and Connon, of Leeds.

Hospital Sunday fixture: Twickenham, 24th July.

The Islington Dispensary, which was established as far back as 1821, has just been rebuilt, and now owns very commodious premises in the Upper Street. "Merrie Islington" has certainly not been without its suffering in the past, for we learn that upwards of half a million patients have been relieved since its inauguration. Last year the dispensary was attended by 15,128 patients.

The other day a very remarkable instance of the value of "first aid," in connection with the St. John's Ambulance Association, occurred. The surgeon lecturer of a Lancashire ambulance class, accompanied by his groom, was himself thrown out of his trap, sustaining a comminuted fracture of the thigh. The groom was also injured. Both were found by some of the pupils of the class, who, after rendering "first aid," conveyed them to the nearest town—Birkdale—where they received medical assistance.

A jubilee appeal has been issued on behalf of the North London Hospital for Consumption, Mount Vernon, Hampstead, for funds to enable the management to pay off the mortgage of £5,000 upon the property. The interest upon this mortgage is a severe drain upon the resources of the institution, and one generous friend has offered to give £1,000 if the remaining £4,000 can be collected during the present year. The committee make themselves responsible for £500, and earnestly appeal to all friends and supporters to second their efforts in this regard.

The Committee of Managers of the Isle of Wight Infirmary are pleading earnestly for increased support. Some time since, through the liberality of special donors, the managers were able to open twelve extra beds, which have since been constantly tenanted. "The Garden of England" has unfortunately its quota of sick poor, who seek the friendly walls of this institution from all parts of the island. The threatened closure of these extra beds, it is therefore to be hoped, will be averted. If each inhabitant of the Isle of Wight would but give the widow's mite, the Ryde Infirmary would be in easy circumstances.

The Chancellor of the Exchequer the other evening made a powerful appeal on behalf of University College Hospital at the festival dinner held at the Criterion. The appeal was liberally responded to, for Mr. Newton H. Nixon, the Secretary, was able to announce, before the conclusion of the proceedings, subscriptions amounting to £1,885. Mr. Goschen observed that he regarded such splendid institutions as University College Hospital as an honour to the metropolis. He spoke in terms of warm praise of the People's Contribution Fund, a special fund to which working men, who are represented among the governors, subscribe. He stated that in the course of the next few years it is hoped to rebuild the hospital, at a cost of £40,000, on a more adequate plan.

THE following vacancies are announced this week, the amount of the salary being placed in each case after the name of the institution :—

**Resident Medical Officers.**

Brighton County Hospital. Assistant. Doubly-qualified.—No salary.
Brompton Cancer Hospital. Assistant House Surgeon. Qualified.—£35.
Devonport Royal Albert Hospital. Assistant House Surgeon. Qualified.—No salary.
Gloucester General Infirmary, House Surgeon. Doubly-qualified.—£100.
National Hospital for the Paralysed. Senior and Junior House Physicians. Doubly-qualified.—£100 and £50 respectively.
Stone Lunatic Asylum, Dartford. Medical Superintendent. Doubly-qualified.—£500.

**Non-resident Medical Officer.**

London Throat Hospital. Dentist.

**Matrons, etc.**

Great Northern Central Hospital.—£60.
St. Pancras Infirmary. Superintendent Nurse.—£50, with allowances.

**Trained Nurses.**

Barnsley Fever Hospital. One.—£45 10s., without board.
Bedford Infirmary. One.—£22.
Bridgewater Infirmary. Night.—£20.
Rotherham Hospital. One.
Royal Albert Asylum for Idiots, Lancaster. One.—£20.
Workington Infirmary. One.—£20.

---

THE British Ophthalmic Hospital and Hospice of the Order of St. John of Jerusalem, was established in the Holy City by the English branch of the Order of St. John in 1882, and is under the patronage of the Prince of Wales. Diseases of the eye largely prevail amongst the poor of Jerusalem and the adjacent country, a fact probably well known to most of our readers, as it has been commented upon by nearly all writers on the subject of Eastern travel. In consequence of good work already done by the hospital, the Sultan of Turkey has accorded his special protection to the institution, and through the Governor of Jerusalem, Raouf Pasha, has contributed towards the purchase of a suitable site and buildings. Mr. Ogilvie, the resident surgeon, is specially skilled in the treatment of ophthalmic disease, and a very large number of operations have been performed during the past year. 121 in-patients and 3,238 out-patients were treated in the hospital during the twelve months ending June last, and out of 231, no less than 132 were what are known as *major* operations. Unfortunately, there are at present only four beds, and the medical report states that on various occasions it has been necessary to admit unusually grave cases of disease, and place the patients on the floor. Several times there have been as many as eleven in-patients at once. The charity is entirely non-sectarian, and sufferers of all religious denominations, Protestants, Greeks, Armenians, Latin Christians, Jews, and Mahommedans, have all been received without distinction. There is, however, no endowment or financial reserve of any kind, and funds to carry on this eminently useful and admirable work are urgently needed. Mr. Moore, the British Consul at Jerusalem, is one of the members of the local committee, and the American Consul, Mr. Merrill, has given his support to the scheme. Any subscriptions or donations, of whatever amount, may be sent to Mr. J. H. Easterbrook, 26, Wilkinson Street, London, S.W., and will be gratefully acknowledged.

WE are glad to notice that our contemporary, *Charity*, has improved in every way since it was taken over by Messrs. Wyman and Sons, who have a deservedly high reputation for newspaper work. Under the editorship of Mr. John Southward, several new features have been introduced into *Charity*, and an abstract of the reports with a summary of the proceedings at the annual meetings of various charities are given each month. *Charity* is published monthly, price sixpence.

---

THE Duke of Cambridge, presiding the other evening at the Hôtel Métropole at the forty-second anniversary dinner of the German Hospital, made an excellent appeal on behalf of this useful and important institution. The Duke has so often pleaded the cause of our hospitals that he has, as he humorously put it, become quite hard-hearted in performing the not too pleasant task of extracting as much money as possible from a gathering of gentlemen. During the evening the secretary announced donations and subscriptions to the amount of £3,409, including £200 from the Emperor of Germany, £50 from the Emperor of Austria, £20 from the Chairman, and £105 from the Corporation of the City of London. The German Hospital is now in a very efficient state, and, in conjunction with the Eastern and Western Dispensaries, which are affiliated to it, relieved last year no less than 26,074 patients, a very large proportion of whom were English.

---

AN appeal, signed by Dr. W. M. Ord, of St. Thomas's; Dr. J. C. Steele, the Superintendent of Guy's ; Dr. W. H. Broadbent, of St. Mary's ; Dr. Symes Thompson, of the Brompton ; Dr. Stephen Mackenzie, of the London ; Mr. A. P. Gould, of the London Temperance Hospital ; Mr. F. M. Corner, Medical Officer of Health for Poplar; and Mr. A. G. Bateman, has been published in the daily papers, calling on the benevolent to assist the Charity Organisation Society with their contributions, for the purpose of enabling that Association to send poor patients to convalescent homes. The signatories state that help has been quickly provided on a plan satisfactory alike to the homes and the patients, and that in many cases when it has been the wage-earner who has been stricken down with illness, pecuniary help has been given to the family. Last year, 2,207 poor patients were sent to convalescent institutions through the instrumentality of this Society, and this year probably more will be restored to health in this way. It would be well if every hospital had that admirable adjunct, a convalescent home for restoring to complete health those whom disease has left enfeebled and low. The period immediately following upon the discharge of the patient from hospital is too often, in the homes of the poor, one of great danger. The enforced absence of probably the bread-winner has reduced the family to abject need, and so on the morrow of his quitting the hospital he is again at his toil, with all the worries and anxieties incidental to life. It is small wonder if the poor man drags out an existence of shattered health, if he does not break down altogether. The Charity Organisation Society has proved in the past such a useful agency in the work of help in convalescence, that we hope the £1,000 for which it asks will soon be raised.

The following sums have been recently received by th various Medical Charities, the name of the donor or the source from which they are obtained being given in each instance after the name of the Institution :—

**Donations.**

British Home for Incurables, Putney. Mr. Wilfred Peek, £15 15*s*. Mr. Stephenson Clarke, 20 guineas.
Charing Cross Hospital. Company of Bowyers, £10 10*s*. Mr. W. H. Yatman, £50.
East London Hospital for Children, Shadwell. Company of Skinners, £10 10*s*.
Evelina Hospital for Sick Children. Company of Skinners, £10 10*s*. Churchwardens of All Hallows, Lombard Street, £10 10*s*.
Hospital for Consumption, St. Leonards-on-Sea. Company of Skinners, £21.
Metropolitan Convalescent Institution, Walton-on-Thames. Company of Skinners, £10 10*s*.
Newmarket and District Hospital. Miss Hodgkinson, £10.
North London Hospital. Mr. G. H. Pinckard, £10 10*s*.
Oldham Infirmary. "A Lady," £300.
Royal United Hospital, Bath. Mr. J. Hopkins, £25. Miss Hopkins, £10. Rev C. B. Barrow, £10. Mrs. Buckle, 10 guineas. Mr. D. C. Commans, 20 guineas. Major Ormond, £53.
St. John's Hospital, Richmond. Mr. Cunard, £50 (Jubilee donation).

**Legacies.**

British Home for Incurables, Putney. Mr. G. H. Johnson, £105.
Carmarthenshire Infirmary. Mr. T. C. Morris, £500.
Chelsea Hospital for Women, Brompton. Mr. G. H. Johnson, £105.
Lincoln County Hospital. Mr. Luke Borman, £50.
Metropolitan Free Hospital. Mr. G. H. Johnson, £105.
Middlesex Hospital. Mr. G. H. Johnson, £105.
North London Hospital, Gower Street. Mr. G. H. Johnson, £105.
Oldham Infirmary. Mr. Redford, £2,706 6*s*. 9*d*.
St. George's Hospital. Mr. G. H. Johnson, £105.
St. Mary's Hospital, Paddington. Mr. G. H. Johnson, £105.
West London Hospital, Hammersmith. Mr. G. H. Johnson, £105.

---

THE Leicester Infirmary is, financially speaking, just now in a most prosperous condition, but this fortunate state of affairs has too much of the element of chance good-luck. The report for the past year, adopted at the annual meeting held recently, showed a handsome donation of £500 from "A Friend," and a legacy of £10,320 from the late Mr. Joseph Poole. Mr. T. F. Johnson, J.P., in seconding the adoption of the report, remarked that, while the receipts showed a surplus over expenditure of £1,009, he wished the causes of the improvement were a little more reliable, for at present their sources of income were, to say the least, precarious. While legacies are always acceptable when they fall in, the benefactions of the living, in the shape of annual subscriptions, are always more to be valued, because they are more stable sources of income. The Leicester Infirmary is well and economically managed, and if its beneficent work were more widely known, the subscribers' list would doubtless be largely extended. A sale of work is shortly to be held, the proceeds of which will be devoted to providing an organ for the chapel and a small library for the nurses. The new wing of the infirmary is now rapidly approaching completion.

---

A SUM of 200 guineas has been voted by the Mercers' Company to the fund for defraying the cost of the structural alterations recently made in the premises of the London School of Medicine for Women, Henrietta Street, W.C. The Clothworkers' Company have again made a grant to the same object, bringing the total they have contributed to this institution up to £190. The school, of which Mrs. Garret Anderson is the dean, is the only recognised medical school in Great Britain which is open to ladies desirous of adopting the profession of medicine. Among other lady doctors educated at this school, who have already greatly distinguished themselves, we may mention Miss Edith Pechey, M.D., and Miss Hitchcock, L.K.Q.C.P.I., who have now responsible positions at Bombay; Miss Edith Shove, M.B., Medical Superintendent of the female staff at the General Post Office, London, and Miss Cradock, L.K.Q.C.P.I., who holds a similar position at the Post Office at Liverpool. Mrs. Scharlieb, M.B., is also an old student of this school.

---

THE past year has been a memorable one in the history of the useful charity known as Queen Charlotte's Lying-in Hospital, by reason of the completion of a new wing, which was formally opened in June last by the Prince and Princess of Wales. In addition to the extra accommodation thus afforded certain alterations of lavatories, etc., have been effected, and the sanitary condition of the whole building greatly improved in consequence. The number of poor women who were benefited by this charity in 1886 was 1,991, 885 of whom were in-patients. Of the latter, eight died in the hospital, but as four of these were extremely serious and complicated cases the death-rate may be considered satisfactorily small. The mortality among the out-patients was under three per 1,000. The total expenditure for the year amounted to £3,804, and the total income to £2,815, so that there was a deficit of £989 demanding the earnest and serious consideration of the management. The Samaritan Fund for the benefit of poor patients leaving the hospital is also inadequate to the demands made upon it, and gifts of old linen and clothing are earnestly solicited by the committee, and may be sent to Mr. Thomas Ryan, the able and energetic secretary, Marylebone Road, W.

---

THE City of London Truss Society not only provides poor persons suffering from rupture and similar affections with the necessary trusses and instruments, but performs operations, and administers surgical aid to those who require it. Moreover, this useful charity is not confined to the metropolis, but extends its benefits to sufferers throughout the kingdom, and since its foundation in 1807 by the late Mr. John Taunton, M.R.C.S., has done an incalculable amount of good and useful work. The 80th annual meeting was recently held. The secretary, Mr. John Whittington, states, the gross yearly income of the institution is subject to great fluctuations, and during 1886 it fell short of the gross expenditure by about £750. This deficiency was due to a falling off in the legacies received, but we are glad to note that the income from subscriptions and donations has been fairly maintained during the past year. The total expenditure amounted to £4,926, and in order to meet the requirements of the charity it was decided at the meeting to sell out £1,250 of the funded property. The necessity for this step is much to be deplored, and we hope that the managers may see their way to prevent its repetition by making special efforts during the current year.

